# Antibiotic-free short-term storage of canine sperm at 5 °C preserves functional and mitochondrial integrity

**DOI:** 10.3389/fvets.2026.1774926

**Published:** 2026-03-19

**Authors:** Macarena Castro, Nicole Carrasco-Zambrano, Felipe Pezo, María José Contreras

**Affiliations:** 1 Doctorado en Ciencias Aplicadas, Facultad de Ingeniería, Universidad Autónoma de Chile, Temuco, Chile; 2 Instituto de Ciencias Aplicadas, Facultad de Ingeniería, Universidad Autónoma de Chile, Temuco, Chile; 3 Facultad de Ciencias Agropecuarias y Medioambiente, Universidad de la Frontera, Temuco, Chile; 4 Centro de Excelencia en Biotecnologías Reproductivas, Universidad de la Frontera, Temuco, Chile

**Keywords:** antibiotic-free extender, canine sperm, hypothermic storage, mitochondrial membrane potential, oxidative stress, refrigerated storage, sperm quality, Tris–egg yolk

## Abstract

The routine prophylactic use of antibiotics in sperm preservation for reproductive biotechnologies poses increasing concerns due to its potential role in promoting antimicrobial resistance. Therefore, developing storage systems that maintain sperm quality without antibiotics is essential. In dogs, chilled sperm is vital in assisted reproduction for genetic improvement and breed conservation. Typically, a 20% Tris–egg yolk extender with broad-spectrum antibiotics supports semen preservation for up to 3 days. This study evaluated a Tris–egg yolk medium without antibiotics, stored at 5 °C, as an alternative for short-term preservation of canine sperm. Eight ejaculates from dogs of different breeds were divided into two aliquots: a control group diluted in Tris–egg yolk with ampicillin, and a treatment group without antibiotics. Samples were stored at 5 °C and analyzed at 0, 24, and 48 h to assess motility, plasma membrane and acrosome integrity, intracellular oxidative stress, and mitochondrial membrane potential. No significant differences were found between treatments in most quality parameters during storage. However, storage time negatively affected membrane and acrosome integrity, as well as mitochondrial function, while slightly increasing oxidative stress. These results indicate that, under controlled short-term refrigeration conditions, canine sperm diluted in a Tris–egg yolk extender without antibiotics maintains functional parameters comparable to antibiotic-supplemented media for up to 48 h, despite time-dependent sub lethal cellular alterations.

## 1 Introduction

Artificial insemination (AI) has become a strategic tool in canine reproduction, particularly within breeding programs and genetic conservation initiatives (1–3). Advances in sperm preservation techniques and the expansion of the global canine industry have positioned AI as a key approach for genetic improvement, disease control, and international germplasm exchange. Among available preservation methods, chilled sperm has gained increasing relevance due to its favorable reproductive outcomes, high fertility efficiency, reduced sanitary risks, and lower stress associated with animal handling, as it only requires the transport of sperm from the donor male to the recipient female (4). Additional advantages include ease of processing and shipment, relatively low costs due to the absence of cryogenic equipment, and fewer legal and logistical constraints compared with frozen sperm (5, 6). When appropriate protocols for sperm collection, preservation, and insemination are applied, the use of chilled sperm has been associated with pregnancy rates and litter sizes equal to or higher than those obtained with frozen sperm (7–9), supporting its widespread use in clinical canine reproduction.

Despite these advantages, the application of chilled sperm is constrained by the limited lifespan of canine spermatozoa during hypothermic storage. Under optimal conditions, refrigerated sperm may retain membrane integrity and motility for 5–7 days (2), and in some cases for more than 10–14 days (10, 11); however, fertilizing capacity declines progressively with storage duration, and insemination is generally recommended within 24–48 h (12, 13). This limitation is particularly relevant considering the unique reproductive physiology of the bitch. Ovulation occurs approximately 48–60 h after the luteinizing hormone surge, but oocytes are released as primary oocytes and require an additional 2–3 days of maturation within the oviduct before becoming fertilizable (14, 15). As a result, successful AI depends on precise synchronization between sperm viability and oocyte maturity. Refrigeration at 5 °C induces a hypometabolic state in spermatozoa, characterized by reduced mitochondrial activity and ATP consumption, which supports short-term survival. However, prolonged hypothermic exposure may promote sublethal cellular stress, including membrane destabilization, mitochondrial dysfunction, and oxidative imbalance, ultimately compromising sperm longevity and functional competence.

Bacteriospermia of canine ejaculates represents an additional challenge during sperm storage. Bacteriospermia has been associated with reduced sperm motility, compromised membrane and acrosomal integrity, increased oxidative stress (ROS), and a higher incidence of morphological abnormalities (16, 17). Microorganisms commonly isolated from sperm of clinically healthy dogs include *Escherichia coli*, *Pasteurella multocida*, *β*-hemolytic *Streptococcus*, *Staphylococcus* spp., *Klebsiella* spp., and *Pseudomonas* spp. (16, 18, 19), while specific pathogens such as β-hemolytic *Streptococcus* spp. have been linked to teratospermia (20). To control bacterial growth, antibiotics are routinely added to sperm extenders (21, 22). However, the systematic use of antimicrobials has contributed to the emergence and dissemination of antimicrobial resistance (AMR), recognized as a global threat to animal and public health (23). In canine reproduction, exposure of both spermatozoa and the female reproductive tract to antibiotics may promote the selection and persistence of resistant bacterial populations, which can subsequently be disseminated into the environment (24). Evidence from livestock systems further indicates that antimicrobial use is associated with increased abundance of resistance genes, highlighting the urgency of antimicrobial stewardship across animal production systems (25).

Despite the widespread incorporation of antibiotics into canine sperm extenders (26–28), evidence regarding the ability of canine spermatozoa to maintain functional competence under antibiotic-free hypothermic storage remains limited. Most studies evaluating chilled sperm preservation rely on extenders containing antimicrobials, making it difficult to distinguish the effects of refrigeration from those attributable to antibiotic supplementation (21, 22). Moreover, although the detrimental effects of bacteriospermia on sperm quality are well documented (16, 17), the duration over which key functional parameters such as membrane integrity, acrosomal status, mitochondrial activity, and oxidative balance can be preserved at 5 °C in the absence of antibiotics has not been clearly defined. This lack of standardized information limits the development of antibiotic-free preservation protocols and perpetuates routine antimicrobial use, despite growing concerns regarding AMR and environmental dissemination of resistance genes (23–25). Therefore, the present study aimed to compare the short-term preservation of canine sperm stored at 5 °C in a Tris–egg yolk extender with or without antibiotics, focusing on functional, membrane, acrosomal, oxidative, and mitochondrial parameters as indicators of sperm viability.

## 2 Materials and methods

The study was conducted at the Universidad Autónoma de Chile, Temuco campus. In the Applied Sciences Laboratory. All experimental procedures and protocols described herein were reviewed and approved by the Scientific Ethics Committee of the Universidad Autónoma de Chile. Procedures were conducted in compliance with the provisions of the Chilean Animal Protection Act (Law N° 20,380). All individuals included in the present study were authorized for these purposes through informed consent obtained from their respective owners.

Unless otherwise specified, all reagents were purchased from Sigma (St. Louis, MO, United States). All solutions were prepared using ultrapure water obtained from a Milli-Q Synthesis system (Millipore, Bedford, MA, United States).

### 2.1 Animals

Eight fertile adult dogs, aged between 3 and 6 years, were used in this study. All dogs varied in size and breed (*n* = 8). Prior to the start of the experiment, each male was clinically examined by a veterinarian to ensure an optimal health and welfare status.

### 2.2 Sperm collection

Sperm samples were collected manually by a veterinarian through manual stimulation, without the need for a female in estrus. The samples were deposited in sterile containers, and the ejaculate volume was recorded. To prevent bacterial contamination from the penile prepuce, the tip of the glans was protruded from the preputial sheath and wiped with sterile gauze prior to collection. Samples were transported to the laboratory in an insulated container within 30 min of collection.

### 2.3 Media

The assays were performed by diluting sperm samples in a 20% Tris–egg yolk extender. The control extender consisted of tris(hydroxymethyl)-aminomethane (3 g), sodium citrate monohydrate (1.7 g), fructose (1.25 g), ampicillin (0.2 g), freshly collected egg yolk 20% (v/v), and distilled water (100 mL) (45). For the antibiotic-free treatment, the same extender was used without the addition of ampicillin. All media were adjusted to a pH of 7.3 ± 0.1 and an osmolarity of 290–300 mOsm/kg.

### 2.4 Sperm processing

**Figure 1:**
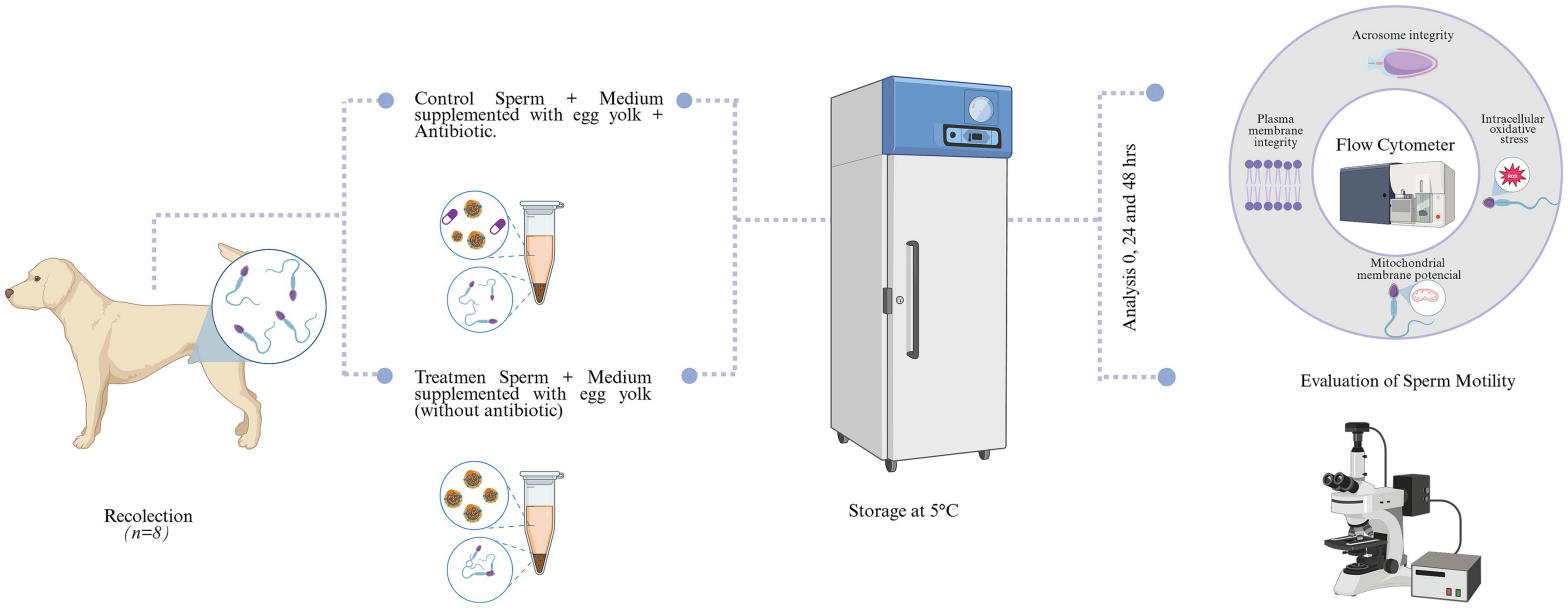
Experimental diagram of canine sperm storage. The sperm was collected and divided into two groups: control (medium supplemented with egg yolk and antibiotic) and treatment (medium supplemented with egg yolk without antibiotic). The samples were stored at 5 °C for 48 h and then analyzed by flow cytometry to assess acrosomal integrity, plasma membrane integrity, mitochondrial membrane potential, and indicators of intracellular oxidative stress at 0, 24, and 48 h. Created in BioRender.com.

After collection, the volume of each ejaculate was measured and recorded in graduated tubes (mL). The sperm concentration was determined using a Neubauer chamber. Each ejaculate was split into two aliquots to ensure a paired experimental design, minimizing inter-individual variability between treatments. Each ejaculate was then centrifuged at 800 × g for 5 min to remove seminal plasma in order to standardize extender composition across treatments and minimize variability associated with endogenous seminal plasma components, acknowledging that this procedure may induce sublethal cellular stress. The sperm pellet was resuspended in the corresponding extender to reach a final concentration of 2 × 10⁶ spermatozoa/mL. Two treatments were prepared: the control group, which used the Tris–egg yolk extender containing ampicillin, and the treatment group, which used the same extender but without antibiotics. Finally, the vials were stored at 5 °C for 2 days. Sperm evaluations were performed at three time points: immediately after collection (fresh sperm; T0), after 24 h (T24), and after 48 h (T48) of storage at 5 °C (Figure 1).

### 2.5 Flow cytometry analysis

All fluorescence analyses were performed using a Becton, Dickinson and Company (BD) FACSAria flow cytometer operated with FACSDiva™ software v6.1.3 (Becton, Dickinson and Company, San Jose, CA, United States). A total of 10,000 events were acquired for each assay, with a flow rate of 60 μL/min. Fluorophores were excited at 488 nm using an argon laser. Green fluorescence was detected using a 530/30 nm bandpass filter, and orange fluorescence was detected using a 585/42 nm bandpass filter. All samples were stained with Hoechst 33342, a DNA-intercalating dye, to ensure that only cells containing DNA, i.e., sperm were analyzed. This allowed exclusion of debris, non-sperm cells, or other particles, ensuring that the population analyzed corresponded specifically to sperm cells.

#### 2.5.1 Evaluation of membrane integrity

Sperm membrane integrity was assessed by using the LIVE/DEAD sperm viability kit (Molecular Probes, Eugene, OR, United States). Aliquots of 500 μL of sperm suspension (2 × 10^6^ sperm/mL) were incubated with 1 μL of 0.2 μМ SYBR 14, 1 μL of 2.4 mM Propidium Iodide (PI) and 1 μL of 845 μМ Hoechst will be added which will be incubated for 15 min in the dark at room. Spermatozoa were analyzed by flow cytometry and classified as previously described.

#### 2.5.2 Evaluation of acrosome integrity

Acrosome membrane integrity was evaluated by using Lectin PNA from *Arachis hypogaea* (peanut) conjugated with FITC (PNA). Aliquots of 500 μL of sperm suspension (2 × 10^6^ sperm/mL) were incubated with 2.5 μL of 1 mg/mL PNA, 1 of 2.4 mM PI and 1 μL 845 μМ Hoechst was added which will be incubated for 15 min. This staining technique led to the identification of four different sperm populations: (16) non viable spermatozoa with intact acrosome (PNA - / PI +), (29) non-viable spermatozoa with reacted acrosome (PNA + / PI +), (30) viable spermatozoa with intact acrosome (PNA - / PI -) and (31) viable spermatozoa with reacted acrosome (PNA +/PI -) (32). The results were expressed as the percentage of viable spermatozoa with reacted acrosome.

#### 2.5.3 Evaluation of ROS

Dihydroethidium (DHE) is a probe specific for the superoxide anion (O₂^−^). Once inside the cell, DHE is oxidized by intracellular superoxide to ethidium, which intercalates into DNA and emits red fluorescence. Aliquots of 500 μL of sperm suspension (2 × 10⁶ sperm/mL) were incubated with 1 μL of 1 mM DHE, 1 μL of 0.06 μM SYTOX Green, and 1 μL of 845 μМ Hoechst. Samples were incubated for 15 min at room temperature in the dark.

#### 2.5.4 Evaluation of mitochondrial membrane potential

Mitochondrial membrane potential was assessed using the MitoProbe™ TMRM assay kit containing tetramethylrhodamine methyl ester (TMRM). Aliquots of 500 μL of sperm suspension (2 × 10⁶ sperm/mL) were incubated with 1 μL of 250 μМ TMRM, 1 μL of 0.06 μM SYTOX Green, and 1 μL of 845 μМ Hoechst. Samples were incubated for 15 min at room temperature in the dark.

### 2.6 Evaluation of total sperm motility

Total motility was analyzed at 0, 24 and 48 h of incubation in the different treatments by taking 10 μL drops of sperm suspension loaded onto a prewarmed slide at 38.5 °C. The drops were covered with coverslips, and the percentage of total motility was estimated under a bright field microscope at 200 × magnification.

### 2.7 Bacteriological limitation of the study

No bacteriological analyses (CFU counts, PCR, or microbiological cultures) were performed in this study. Therefore, the conclusions are restricted to the evaluation of sperm functional and physiological parameters during short-term storage and do not address microbial safety or bacterial control.

### 2.8 Statistical analysis

Data were analyzed using descriptive statistics based on the mean and standard error (SE) calculated for each variable using StatGraphics Plus 5.1 (StatPoint Technologies Inc., Warrenton, VA, United States). Quantitative data were analyzed using analysis of repeated-measures variance (ANOVA). *Post hoc* multiple comparisons using Tukey’s test were performed only when a significant main effect was detected. Differences were considered statistically significant when *p* < 0.05.

Regarding sample size and statistical justification, the study was based on eight ejaculates and a *post hoc* power analysis was conducted for the primary outcome of interest, corresponding to the row factor (storage time) in the ANOVA. The analysis showed a highly significant effect of time [*F*(2, 29) = 44.19], with a very large effect size (partial eta squared = 0.71; Cohen’s *f* = 1.57). With a significance level of *α* = 0.05, the achieved statistical power was greater than 0.99.

## 3 Results

### 3.1 Effects of sperm treatments on sperm plasma membrane integrity

**Figure 2:**
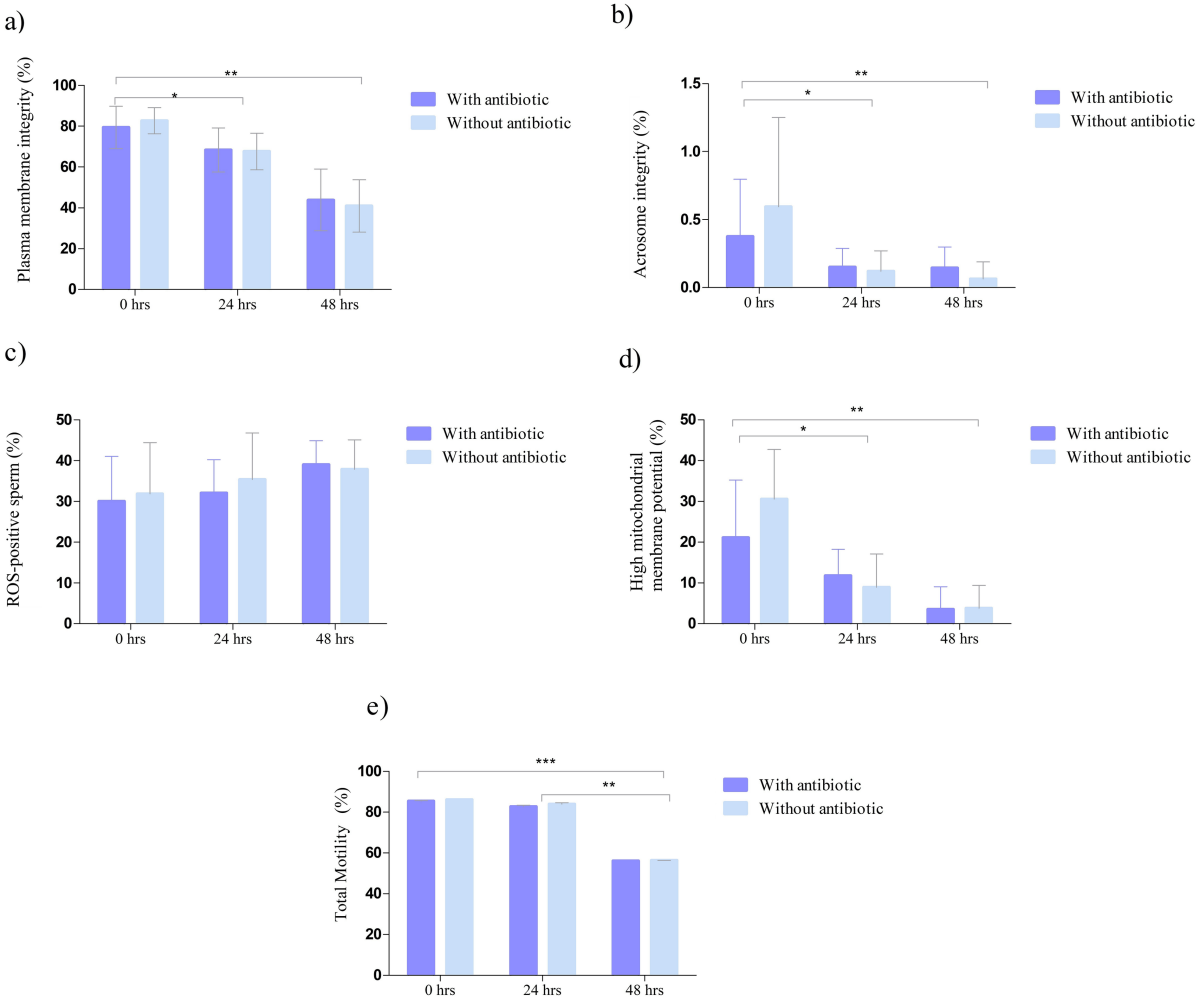
Evaluation of functional and physiological parameters of canine sperm stored at 5 °C in egg yolk–supplemented medium, with antibiotic (dark blue) and without antibiotic (light blue), at 0, 24, and 48 h of storage: **(a)** Plasma membrane integrity (%); **(b)** acrosome integrity (%); **(c)** oxidative stress levels (%); **(d)** mitochondrial membrane potential (%); and **(e)** total sperm motility (%). Values are expressed as mean ± standard error of the mean (SEM). Brackets indicate significant differences between incubation times according to Tukey’s *post hoc* test (*p < 0.05, **p < 0.01, ***p < 0.001).

ANOVA revealed a significant effect of storage time on sperm plasma membrane integrity [F(2, 29) = 44.19, *p* < 0.0001]. In contrast, no significant effect of treatment was observed (*p* = 0.9697), nor was there a significant Time × Treatment interaction (*p* = 0.7594). *Post hoc* multiple comparisons using Tukey’s test, applied only for the significant main effect of time, indicated that sperm plasma membrane integrity at 0 h was significantly higher than at 24 h and 48 h (*p* < 0.05) (Figure 2A).

### 3.2 Effects of sperm treatments on acrosome integrity

ANOVA revealed a significant effect of storage time on acrosome integrity assessed by PNA staining [*F*(6, 29) = 6.249, *p* = 0.0042]. In contrast, no significant effect of treatment was observed (*p* = 0.7190), nor was there a significant Time × Treatment interaction (*p* = 0.4139). *Post hoc* multiple comparisons using Tukey’s test, applied only to the significant main effect of time, indicated significant differences between incubation times (*p* < 0.05) (Figure 2B).

### 3.3 Effects of sperm treatments on ROS

ANOVA revealed no significant effect of storage time on intracellular ROS levels assessed by DHE [*F*(6, 29) = 2.434, *p* = 0.0999]. Likewise, no significant effect of treatment was observed (*p* = 0.6361), nor was there a significant Time × Treatment interaction (*p* = 0.8058) (Figure 2C).

### 3.4 Effects of sperm treatments on mitochondrial membrane potential

ANOVA revealed a significant effect of storage time on mitochondrial membrane potential assessed by TMRM [*F*(6, 29) = 24.06, *p* < 0.0001]. In contrast, no significant effect of treatment was observed (*p* = 0.3987), nor was there a significant Time × Treatment interaction (*p* = 0.1616). *Post hoc* multiple comparisons using Tukey’s test, applied only for the significant main effect of time, indicated significant differences between incubation times (*p* < 0.05) (Figure 2D).

### 3.5 Effects of treatments on sperm motility analysis

ANOVA revealed a highly significant effect of storage time on total sperm motility [*F*(6, 29) = 2,913, *p* < 0.0001]. In contrast, no significant effect of treatment was observed (*p* = 0.0501), nor was there a significant Time × Treatment interaction (*p* = 0.5071). Post hoc multiple comparisons using Tukey’s test, applied only for the significant main effect of time, revealed significant differences between incubation times (*p* < 0.05) (Figure 2E).

## 4 Discussion

In the present study, the feasibility of preserving canine spermatozoa at 5 °C for 48 h in a Tris–egg yolk extender. The results indicate that, under aseptic handling conditions, the omission of antibiotics does not significantly compromise spermatozoa quality during the first 48 h of storage, as no statistically significant differences were observed between treatments for most evaluated parameters. This finding supports the hypothesis that cellular deterioration observed in refrigerated spermatozoa is mainly due to physical and metabolic stress induced by low temperatures, rather than active bacterial proliferation in the short term, especially when starting from samples with controlled microbial load.

Previous studies in dogs, such as those by Domrazek et al. (33), have shown that the presence of certain microorganisms in the ejaculate does not always correlate with negative changes in basic spermatozoa parameters, such as motility and cellular integrity, which is consistent with the findings of the present study. However, no significant differences in mitochondrial stability were observed between the groups that received antibiotic supplements and those that did not, which could be more relevant in studies with larger samples or longer storage periods. Overall, time was identified as the main factor in progressive loss. Overall, time was identified as the primary factor in the progressive loss of spermatozoa quality, negatively affecting plasma membrane integrity, acrosomal integrity, total motility, and mitochondrial potential after 24 h of storage. Total motility decreased from 86% at 0 h to 83% at 24 h and 53% at 48 h. This aligns with reports in the literature regarding the effects of refrigeration on canine spermatozoa physiology (4, 34). In particular, the decrease in mitochondrial membrane potential observed as early as 24 h likely reflects an early hypometabolic shift induced by refrigeration, characterized by reduced ATP production. This metabolic downregulation precedes overt motility loss and may serve as an early indicator of progressive functional exhaustion during storage. (29).

To interpret these time-dependent changes fully, the physiological mechanisms governed by hypothermia must be considered. The cooling process triggers a phase transition in the sperm plasma membrane lipids from a liquid-crystalline to a gel state (35, 36). This transition creates structural instabilities and alters the selective permeability of the membrane, potentially disrupting the function of ion channels and osmotic regulation mechanisms (37). Simultaneously, while storage at 5 °C induces metabolic quiescence, it also imposes stress on mitochondrial bioenergetics. The observed decline in mitochondrial membrane potential after 24 h suggests that hypothermia may cause a gradual uncoupling of the electron transport chain (31). Furthermore, although metabolic rates are reduced, the electron transport chain continues to generate reactive oxygen species (ROS) at a basal level. In the absence of optimal enzymatic antioxidant activity, this can lead to sublethal oxidative stress (30, 38), contributing to the functional decline observed.

Regarding ROS, no differences were observed between treatments or significant changes over time, suggesting that the Tris–egg yolk extender provides sufficient antioxidant capacity to mitigate the generation of reactive oxygen species (ROS), even in the absence of antibiotics. This aspect is particularly relevant considering that one of the main arguments for prophylactic antimicrobial use in preservation media is to prevent increased oxidative stress resulting from bacterial proliferation. Studies in other species, such as pigs, have also observed that exclusion of antibiotics does not significantly affect spermatozoa quality during the first few days, although bacterial growth increases from the second day onward (39). However, storage at 5 °C has been shown to efficiently control bacterial growth, suggesting that this refrigeration strategy could serve as an optimal method of control without the need for antibiotics. This alternative allows for the preservation of spermatozoa quality while minimizing the risk of bacterial resistance associated with prophylactic antimicrobial use.

From the perspective of the global antimicrobial resistance (AMR) problem, these results are encouraging, as they suggest that it is possible to reduce antibiotic use in artificial insemination programs without compromising spermatozoa quality, provided that rigorous hygiene practices are adopted. Domrazek et al. (33) emphasize that indiscriminate antibiotic use can alter the reproductive microbiota and promote the selection of resistant strains, which is relevant from both clinical and ecological perspectives. Nevertheless, the omission of antibiotics is safe only during the first 48 h of storage at 5 °C, provided that strict hygiene conditions are maintained and an appropriate extender is used. Exceeding this period without antibiotics could favor bacterial growth, leading to uterine infections, fertilization failure, embryonic or fetal resorption, abortions, or stillbirths, contributing to smaller litter sizes and, in severe cases, septicemia in the female (40). Therefore, extending storage beyond 48 h without antimicrobials could represent a serious error, both due to spermatozoa deterioration and potential negative effects on female reproductive health.

A complementary strategy for reducing antibiotic use in spermatozoa preservation involves good hygiene practices and processing techniques. Procedures such as single-layer colloid centrifugation and filtration methods constitute effective tools for decreasing bacterial contamination in spermatozoa samples without resorting to routine antimicrobials. These methodologies allow for the separation of healthy spermatozoa from bacteria, cellular debris, and part of the seminal plasma, improving spermatozoa quality and reducing the risk of microbial proliferation during storage (41, 42, 43). Their implementation has been consolidated as a valid alternative within strategies aimed at reducing selective pressure associated with antibiotic use in reproduction, allowing for the maintenance of adequate spermatozoa parameters without compromising the biosafety of insemination doses (43, 44).

Although refrigeration at 5 °C is known to slow bacterial proliferation, the absence of direct bacteriological measurements in this study limits conclusions to sperm physiological performance rather than microbial control. Despite the robustness of the results, this study has several limitations that should be considered as opportunities for future research. The bacterial load of the samples was not quantified during storage, preventing determination of whether subclinical bacterial growth occurred, which, although it did not affect the functional parameters measured, could have implications for reproductive health or pathogen transmission. Future studies could incorporate quantitative microbiological analyses, both aerobic and anaerobic, as well as molecular techniques such as PCR or sequencing to identify and monitor changes in bacterial load. It is also important to emphasize that these findings are preliminary, and further research is needed to comprehensively evaluate the reproductive impact of antibiotic-free extenders. This includes *in vitro* fertilization assays, field fertility trials, assessment of pregnancy rates, and monitoring of litter size, among other relevant outcomes. Therefore, the present study does not suggest that antibiotics should be omitted in practice but rather represents a first step toward developing and validating an alternative preservation strategy. From a practical perspective, sperm preservation without antibiotics emerges as a viable and potentially sustainable approach, with the potential to reduce costs, minimize selective pressure on bacterial resistance, and simplify extender formulation, provided that biosafety standards are maintained. Taken together, the lack of fertility trials and bacteriological quantification constrains the extrapolation of these findings to routine clinical practice; the results should be interpreted as preliminary evidence of short-term sperm tolerance rather than definitive proof of biosafety. Microbiological assessments and *in vivo* fertility trials are required before such protocols can be safely recommended for routine clinical application.

## 5 Conclusion

The results of this study demonstrate that the Tris–egg yolk 20% extender without antibiotics can preserve the functional parameters of canine sperm for up to 48 h at 5 °C under controlled laboratory conditions. It is important to emphasize that this observed effectiveness refers to short-term *in vitro* sperm functionality and does not directly reflect reproductive outcomes or microbiological safety. The study did not quantify microbial load, did not assess *in vivo* fertility, and was conducted on a relatively small sample size; therefore, extrapolation to routine clinical practice should be made with caution.

Although antibiotic-free extenders have the potential to reduce unnecessary antimicrobial use and may indirectly contribute to mitigating antimicrobial resistance, this effect was not directly demonstrated in the present study. From a practical standpoint, sperm preservation without antibiotics represents a viable and potentially sustainable approach under strictly controlled hygienic conditions, but its applicability remains limited to such environments until further validation is performed. Future research should include microbial load analyses, in vivo fertility trials, and larger sample sizes to comprehensively assess reproductive performance and biosafety, thereby supporting responsible implementation in clinical reproductive programs.

## Data Availability

The raw data supporting the conclusions of this article will be made available by the authors, without undue reservation.

## Supplementary material

SUPPLEMENTARY FIGURE 1 Representative bivariate fluorescence dot plots showing the gating strategy and sperm subpopulations identified by flow cytometry. (A) SYBR-14/propidium iodide (PI) staining showing live sperm (SYBR-14^+^/PI^-^) and dead sperm (SYBR-14^-^/PI^+^). (B) FITC PNA/SYTOX Green staining represents acrosomal integrity. D PNA+ corresponds to dead sperm with damaged or reacted acrosomes, and D PNA- to dead sperm with intact acrosomes, L DNA+ corresponds to live sperm with damaged or reacted acrosomes, and L DNA- to live sperm with intact acrosomes. (C) DHE/SYTOX Green staining represents the presence of reactive oxygen species (ROS). D DHE+ corresponds to dead sperm with ROS presence, and D DHE- to dead sperm with low ROS levels. L DHE+ corresponds to live sperm with high ROS levels, and L DHE- to live sperm with low ROS levels. (D) Tetramethylrhodamine methyl ester (TMRM) and SYTOX™ staining identifying sperm with high mitochondrial membrane potential (TMRM^+^/SYTOX^-^), low mitochondrial membrane potential (TMRM-/SYTOX-) and dead sperm (SYTOX^+^).
