# Supplementary figures and images for: Antibiotic-free short-term storage of canine sperm at 5 °C preserves functional and mitochondrial integrity
Source: Front Vet Sci. 2026 Mar 19;13:1774926. doi: 10.3389/fvets.2026.1774926 (PMC13043342; doi:10.3389/fvets.2026.1774926)

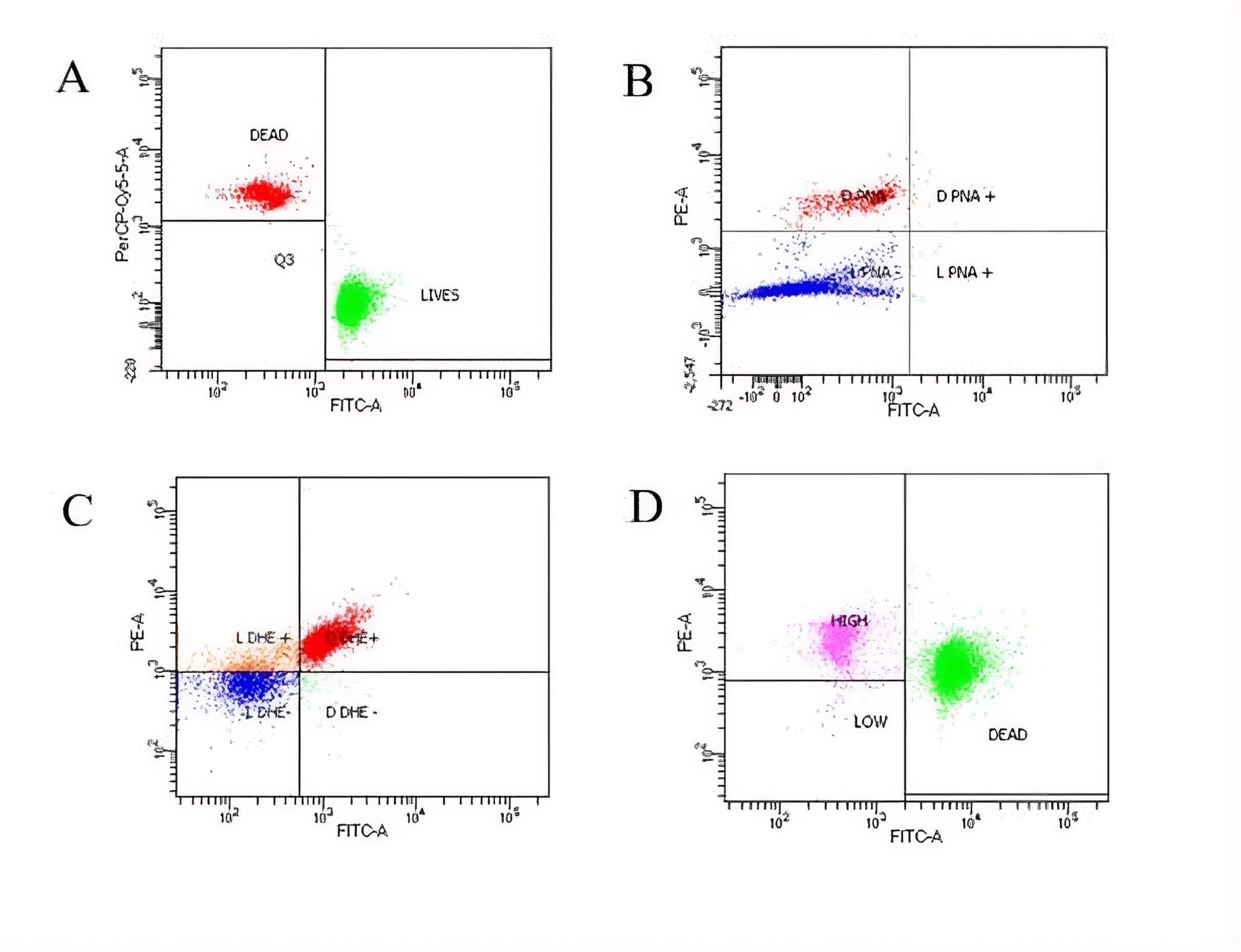

Supplement: SUPPLEMENTARY FIGURE 1 — Representative bivariate fluorescence dot plots showing the gating strategy and sperm subpopulations identified by flow cytometry. (A) SYBR-14/propidium iodide (PI) staining showing live sperm (SYBR-14+/PI-) and dead sperm (SYBR-14-/PI+). (B) FITC PNA/SYTOX Green staining represents acrosomal integrity. D PNA+ corresponds to dead sperm with damaged or reacted acrosomes, and D PNA- to dead sperm with intact acrosomes, L DNA+ corresponds to live sperm with damaged or reacted acrosomes, and L DNA- to live sperm with intact acrosomes. (C) DHE/SYTOX Green staining represents the presence of reactive oxygen species (ROS). D DHE+ corresponds to dead sperm with ROS presence, and D DHE- to dead sperm with low ROS levels. L DHE+ corresponds to live sperm with high ROS levels, and L DHE- to live sperm with low ROS levels. (D) Tetramethylrhodamine methyl ester (TMRM) and SYTOX™ staining identifying sperm with high mitochondrial membrane potential (TMRM+/SYTOX-), low mitochondrial membrane potential (TMRM-/SYTOX-) and dead sperm (SYTOX+). [file Image_1.png]
